# Letter in response to “Vascular endothelial cadherin shedding is more severe in sepsis patients with severe acute kidney injury”

**DOI:** 10.1186/s13054-019-2455-0

**Published:** 2019-05-14

**Authors:** Richard W Pierce

**Affiliations:** Yale University, 333 Cedar Street, P.O. Box 208064, New Haven, CT 06520-8064 USA

Dear Editor,

I read with great interest Yu et al.’s study on vascular endothelial cadherin (VE-cadherin) in patients with sepsis and kidney injury [1]. The endothelium has long been implicated in the pathogenesis of critical illness. Despite our enthusiasm, insights linking changes occurring within endothelial cells (ECs) to clinically observed pathophysiology are few and far between. As noted, VE-cadherin is a complex molecule, a component of adherens junctions (AJs) as well as a multifaceted signaling molecule. Yu et al.’s study highlights the clinical utility of VE-cadherin as a potential biomarker of EC injury but falls short of providing mechanistic insight to EC dysfunction in critical illness.

VE-cadherin is specific to ECs and may be released as soluble-(s)VE-cadherin through several mechanisms, each with distinct pathophysiologic implications. Intact VE-cadherin may detach from the membrane of dead or dying ECs. More commonly, VE-cadherin may be actively cleaved, forming sVE-cadherin species with distinct molecular weights and, presumably, functions [2]. Different cleavage sites for VE-cadherin are identified for specific matrix metalloproteinases; however, no sVE-cadherin species has a known physiologic role [3]. Similarly, soluble adhesion molecules (i.e., sICAM) are commonly studied as markers of EC “dysfunction” but have no understood biologic functions, complicating interpretation of elevated levels. Finally, VE-cadherin may be shed in EC-derived microvesicles, which serve as signaling platforms to other ECs or immune cells [4]. Unfortunately, the study by Yu and colleagues does not demarcate the type of VE-cadherin detected in patient’s blood, complicating interpretation of its mechanistic implications.

Furthermore, the authors expressed surprise over the lack of correlation with sVE-cadherin and total fluid balance. This finding could be explained by the differential junctional specificity across vascular segments. AJs are characteristic of the venular segment. Venular leak is physiologic, allowing for localized inflammatory response and rarely results in clinical consequences. The capillary vascular segment is characterized by tight junctions (TJs). Once established, TJ integrity does not depend on AJs. Capillary leak is pathologic and, due to exponentially greater cumulative surface area, is clinically impactful. TJ breakdown can be assessed in critically ill patients [5], and preserved TJ function may explain the lack of correlation between sVE-cadherin and fluid balance.

Investigations of the vascular response to critical illness remain paramount to our understanding of complex diseases like shock, sepsis, and multiple organ dysfunction. Broadly characterizing vascular components will likely not result in meaningful mechanistic insights. Special attention to the vascular segment and the meaningfulness of surveyed byproducts of EC dysfunction will better advance our field.

Sincerely,

Richard Pierce, MD, MS

Yale University, Department of Pediatrics, Section of Critical Care

Vascular Biology and Therapeutics Program
